# Mutant p53 and sIL-1Ra

**DOI:** 10.18632/aging.100825

**Published:** 2015-10-20

**Authors:** Gianluca Bossi

**Affiliations:** Experimental Oncology Laboratories, and Laboratory of Medical Physics and Expert Systems Regina Elena National Cancer Institute, Rome, Italy

**Keywords:** p53, tumor, microenvironment, inflammation, antagonist receptors, cytokines

The microenvironment of solid tumors is composed of malignant cells surrounded by a reactive stroma containing extracellular matrix with a huge infiltration of non-malignant populations (myeloid cells, lymphocytes, fibroblast, endothelial cells), which together with several cytokines/chemokines, tissue remodelling and angiogenesis support an inflammatory milieu. Tumor growth and metastasis are the result of a complex bidirectional interaction between cells that progressively acquire molecular alterations, and a transformed phenotype (cell-autonomous functions) and the surrounding host cells (non-cell-autonomous functions).

The TP53 gene is mutated in about half of all human cancers. Missense mutations are the most prevalent alterations (75%) located mainly within the DNA binding domain. Most of these alterations hold the full-length protein, often present in grossly elevated levels compared to the wild type (wt) p53 in normal cells, that lose the tumor suppressor functions (loss-of-function, LOF), and acquire novel functions (gain-of-function, GOF) through which contribute to tumorigenesis, tumor progression and chemo- or radiotherapy resistance. In recent years many cell-autonomous GOFs mutant (mut) p53 have been described, mostly linked to the ability of mutated proteins to control the expression of specific target genes [1]. Noteworthy, emerging evidences uncovered the existence of non-cell-autonomous wtp53 functions by promoting anti-tumor microenvironment [2, 3], whereas barely reported the non-cell-autonomous GOFs mutp53 [4].

We previously demonstrated that inducible depletion in vivo of endogenous mutp53 reduces tumor growth, stromal invasion, and angiogenesis in xenografted HT29 colon cancer cells [5]. Founded on these results we enquired whether GOFs mutp53 might be involved in the tumor microenvironment (TME) crosstalk. To this aim we analysed the cytokine secretion profile in a panel of colon and breast human cancer cells, and identified the soluble interleukin-1 receptor antagonist (sIL-1Ra) as a novel mutp53 repressed target gene [6]. The sIL-1Ra is a natural occurring anti-inflammatory cytokine that acts as a specific antagonist of the Interleukin-1 (IL-1) α and β pro-inflammatory cytokines: it binds to both type I and type II IL-1 receptors (IL-1R1 and IL-1RII), with approximately equal affinity as compared with IL-1α and IL-1β, without exerting any agonist activity. The IL-1β is a highly active and pleiotropic pro-inflammatory cytokine implicated in the pathogenesis of many inflammation-associated diseases. Indeed, recombinant sIL-1Ra (Kineret) is currently used to cure a number of inflammatory and orthopaedic disease. Importantly, the IL-1β expression is found elevated in several human tumors (breast, colon, lung, head and neck, and melanomas), and patients with IL-1β producing tumors have generally bad prognosis.

We found that mutp53 represses whereas activated wtp53 induces sIL-1Ra gene expression uncovering a novel GOF mutp53 [6]. Mechanistically, we identify the MAFF as common molecular player in the opposite regulation of sIL-1Ra gene expression by mut and wtp53 [6]. Indeed, the small MAFs abundance has been identified as a fine tuning molecular switch regulating positively or negatively gene expression. To evaluate the biological significance of sIL-1Ra suppression in GOF mutp53, we explored the cancer cell response to recombinant IL-1β along with mutp53 depletion or kineret pre-treatment. Results revealed that similarly to pre-treatment with recombinant sIL-1Ra, the derepressed sIL-1Ra in mutp53 depleted cells, hampers the IL-1β signalling cascade by reducing IL-1 target genes expression in vitro and in vivo; the cancer cell proliferation in vitro; and the growth of xenografted tumor in LPS-treated mice. Additionally, the supernatants of either mutp53 depleted or kineret pretreated cancer cells abolishes the IL-1β-induced HUVEC endothelial cell monolayer permeability, a hallmark of early angiogenesis [6].

The study shown for the first time the existence of a functional link between sIL-1Ra and mutp53, adding further insights for the identification of novel non-cell-autonomous GOFs mutp53 in human cancer. Thus, mutp53 by repressing sIL-1Ra could sustain a prompt IL-1β cancer cell response promoting a chronically inflamed TME, hence fostering further malignancy. Noteworthy, chronic-inflammation is a predisposing cause in various malignancies and is often characterized as the seventh hallmark of cancer [7]. Recent investigations reported that mutp53 sustains cancer progression by augmenting nuclear factor κB (NFκB) activation in the context of chronic inflammation in vitro and in vivo [8]. The NFκB is a required transcription factor for canonical IL-1 target genes expression. Accordingly, we propose that mutp53 might support a ready-to-be-activated IL-1 signalling cascade in cancer cells through a dual regulatory path: - extracellularly by suppressing the sIL-1Ra production, thus reducing the relative protein levels of the receptor antagonist in the microenvironment nearby the cancer cells; - intracellularly increasing the IL-1 target gene expression augmenting NFkB activity.

Remarkably, preclinical studies provide ample support to propose the reduction of IL-1 activity as a potential therapeutic target in human cancers. Accordingly, albeit further investigations are required, achieved results are suggesting that modulation of the TME through the targeting of IL-1 activity combined with currently used chemotherapeutic agents might constitute a novel efficient anti-tumoral strategy for treating mutp53 carrying tumors.

## References

[1] Muller PA, Vousden KH (2013). Nat. Cell Biol.

[2] Lujambio A (2013). Cell.

[3] Bar J (2009). Oncogene.

[4] Addadi Y (2010). Cancer Res.

[5] Bossi G (2008). Cell Cycle.

[6] Ubertini V (2015). Oncogene.

[7] Mantovani A (2009). Nature.

[8] Cooks T (2013). Cancer Cell.

